# Loss of Protein Kinase C-δ Protects against LPS-Induced Osteolysis Owing to an Intrinsic Defect in Osteoclastic Bone Resorption

**DOI:** 10.1371/journal.pone.0070815

**Published:** 2013-08-08

**Authors:** Ee Cheng Khor, Tamara Abel, Jennifer Tickner, Shek Man Chim, Cathy Wang, Taksum Cheng, Benjamin Ng, Pei Ying Ng, Dian Astari Teguh, Jacob Kenny, Xiaohong Yang, Honghui Chen, Keiichi I. Nakayama, Keiko Nakayama, Nathan Pavlos, Ming H. Zheng, Jiake Xu

**Affiliations:** 1 Centre for Orthopaedic Research, School of Surgery, University of Western Australia, Nedlands, Western Australia, Australia; 2 School of Pathology and Laboratory Medicine, University of Western Australia, Nedlands, Western Australia, Australia; 3 Centre for Microscopy, Characterisation and Analysis, University of Western Australia, Nedlands, Western Australia, Australia; 4 Guangzhou Institute of Traumatic Surgery, the Fourth Affiliated Hospital of Medical College, Jinan University, Guangzhou, China; 5 Department of Molecular and Cellular Biology, Medical Institute of Bioregulation, Kyushu University, Fukuoka, Japan; 6 Department of Molecular Genetics, Medical Institute of Bioregulation, Kyushu University, Fukuoka, Japan; University of Illinois at Chicago, United States of America

## Abstract

Bone remodeling is intrinsically regulated by cell signaling molecules. The Protein Kinase C (PKC) family of serine/threonine kinases is involved in multiple signaling pathways including cell proliferation, differentiation, apoptosis and osteoclast biology. However, the precise involvement of individual PKC isoforms in the regulation of osteoclast formation and bone homeostasis remains unclear. Here, we identify PKC-δ as the major PKC isoform expressed among all PKCs in osteoclasts; including classical PKCs (−α, −β and −γ), novel PKCs (−δ, −ε, −η and −θ) and atypical PKCs (−ι/λ and −ζ). Interestingly, pharmacological inhibition and genetic ablation of PKC-δ impairs osteoclastic bone resorption *in vitro*. Moreover, disruption of PKC-δ activity protects against LPS-induced osteolysis in mice, with osteoclasts accumulating on the bone surface failing to resorb bone. Treatment with the PKC-δ inhibitor Rottlerin, blocks LPS-induced bone resorption in mice. Consistently, PKC-δ deficient mice exhibit increased trabeculae bone containing residual cartilage matrix, indicative of an osteoclast-rich osteopetrosis phenotype. Cultured ex vivo osteoclasts derived from PKC-δ null mice exhibit decreased CTX-1 levels and MARKS phosphorylation, with enhanced formation rates. This is accompanied by elevated gene expression levels of cathepsin K and PKC −α, −γ and −ε, as well as altered signaling of pERK and pcSrc416/527 upon RANKL-induction, possibly to compensate for the defects in bone resorption. Collectively, our data indicate that PKC-δ is an intrinsic regulator of osteoclast formation and bone resorption and thus is a potential therapeutic target for pathological osteolysis.

## Introduction

Bone remodeling is a tightly coupled process involving bone-forming osteoblasts and bone-resorbing osteoclasts [1]. Under physiological conditions, osteoblast and osteoclast activity is delicately balanced, thereby maintaining a steady bone mass in young adults. However, an imbalance in bone homeostasis in favor of osteoclast activity can lead to osteolytic diseases including osteoporosis, Paget's disease, and rheumatoid arthritis [2]. It is also commonly observed in systemic or local inflammation in bone, and in cancer cell metastasis to bone that results in hypercalcemia and pathological fractures in affected individuals [2]. A unifying pathological feature among these disorders is elevated osteoclast-mediated bone resorption resulting in net bone loss or osteolysis [3].

Osteoclasts are large polykaryons originating from bone marrow-derived monocyte precursor cells stimulated with Receptor Activator of NF-κB Ligand (RANKL) and Macrophage Colony Stimulating Factor (M-CSF) [4], [5]. RANKL is typically expressed by osteoblasts and binds to its receptor RANK expressed on precursor cells and mature osteoclasts to initiate signals for osteoclastogenesis and bone resorption, respectively [6], [7]. Bone resorption is a specialized function performed by osteoclasts. The process involves cytoskeletal polarization and podosome formation resulting in the formation of the sealing zone and ruffled border which is essential for osteoclastic resorption [3]. Bone resorption is regulated by Src activity [8] together with a cascade of comparatively poorly defined signaling molecules. Therefore, research into the molecular mechanisms underlying osteoclast activity is beneficial in providing new avenues for the prevention and treatment of bone disease.

Protein Kinase Cs (PKCs) are a family of serine/threonine kinases involved in a multitude of cellular processes including cell proliferation, differentiation, apoptosis, and survival [9]–[11]. The multigene family consists of at least 10 members classified into three groups. Classical PKCs (−α, −βI, −βII and −γ) are Ca^2+^, Diacylglycerol (DAG) and phosphatidylserine (PS) dependent. Novel PKCs (−δ, −ε, −η and −θ) are DAG and PS dependent. Finally, the Atypical PKCs (−ι/λ and −ζ) are PS dependent [9], [12]. PKCs are generally involved in cell survival and apoptosis, thus their expression is altered in some forms of cancer [12]. Intriguingly, PKCs are involved in RANKL-induced signaling and osteoclastogenesis [13] and in other signaling pathways associated with osteoclast formation and function [14]–[16]. However, the precise involvement of individual PKC isoforms in the regulation of bone homeostasis and osteoclast function is largely unknown. Considering studies from other cell systems, it is plausible that PKC-δ functions in the regulation of multiple molecular pathways including ERK, NF-κB, p38 MAPK, JNK and Src signaling in both osteoclasts and osteoblasts [10], [17]–[20]. Indeed, a recent study has shown that loss of PKC-δ results in a defect in osteoblast differentiation leading to reduced bone formation during embryonic development [21]. In addition, PKC-δ-deficient mice exhibit an immune disorder with increased proliferation of B cells and auto-immunity in mice [22]. However, the role of PKC-δ in inflammatory-induced osteolysis and in osteoimmunology is hitherto unknown.

Here, we provide evidence that deletion and/or inhibition of PKC-δ protects against LPS-induced osteolysis in mice. Our studies indicate that PKC-δ is the predominant PKC isoform expressed in osteoclasts, and plays an important role in osteoclast bone resorption function, thereby opening up new avenues for pharmaceutical intervention in pathological osteolytic conditions.

## Materials and Methods

### Ethics Statement

All animal handling procedures complied with National Health and Medical Research Council Guidelines and were approved by the Animal Ethics Committee (AEC) (AEC No. 3/100/755) of the University of Western Australia (Perth, Western Australia, Australia). Giant Cell Tumour (GCT) tissue, sourced from 2 independent cases, was collected fresh from patients postoperatively (Sir Charles Gairdner Hospital, Nedlands, WA, Australia). All patients gave written consent, and experiments were approved by the Sir Charles Gairdner Group Human Research Ethics Committee (HREC No. 2011–139).

### Antibodies and reagents

Antibodies for western blotting were purchased from the following sources: mouse IκB-α (C-21), Phospho-ERK1/2 (E-4) (Santa Cruz Biotechnology Inc.); mouse JNK (#9252), Phospho-JNK (#9251), p38 (#9212), Phospho-p38 (#9211), PKC-δ (#2058), Phospho Thr-505 PKC-δ, Phospho-PKC(pan)(#9371), Phospho Tyr-416 Src family (#2101), Phospho-MARCKS (#8722) (Cell Signaling); mouse NFATc1 (556602) (BD Pharmingen); mouse ERK1/2 (V114a) (Promega Corp.); mouse beta-actin (JLA20) (Calbiochem); mouse anti-c-Src antibody was a gift by A/Prof Heung-Chin Cheng, Australia; conjugated peroxidase IgG antibodies and mouse anti-α-Tubulin (T3526) were purchased from Sigma-Aldrich. Rhodamine Phalloidin 546, Alexa Fluor® 647 Phalloidin and Alexa Fluor® conjugated antibodies were purchased from Invitrogen. Antibodies for flow cytometry were purchased from BD Biosciences.

### Generation of PKC-δ Knockout (KO) mice

PKC-δ KO mice were originally generated by Miyamoto *et al* as previously described [22]. Briefly, a targeting vector was used to replace exon I and II of the PKC-δ gene with a PGK-neo-poly(A) cassette in 129/Sv embryonic stem cells (ES). The mutant ES cells were microinjected in to C57BL/6 blastocysts and the resulting male chimeras were mated with female C57BL/6 mice. Heterozygous mice were intercrossed to produce homozygous mice. The mice were backcrossed to a C57BL/6 background for 10 generations.

### Lipopolysaccharide (LPS)-induced osteolysis model

LPS (25 mg/kg) or vehicle was injected subcutaneously into the tissue pocket surrounding the calvaria of four month old PKC-δ KO mice and wild-type (WT) controls. Mice were sacrificed seven days post injection, and the calvaria was removed and fixed in 10% Neutral Buffered Formalin (NBF) for histological examination.

### Histology and Morphometric Analysis

Double-fluorochrome labeling was performed using sterilized calcein (MP Biomedical) and was administered to KO and WT mice by intraperitoneal injection at a dose of 5 mg/kg. A second calcein injection was performed five days after the first injection. Mice were sacrificed two days after the second injection. The hindlimbs from age and sex-matched WT and PKC-δ deficient mice were fixed in 10% NBF, plastic embedded and sectioned. Fluorescence was visualized by confocal microscopy. Interlabel width (μm) between double labels was measured to calculate mineral apposition rate (MAR). Bone histomorphometric analysis of decalcified paraffin-embedded sections stained with haematoxylin and eosin (H&E) and tartrate-resistant acid phosphatase (TRAP) was performed using a Nikon microscope equipped with a digital camera and image analysis software (Osteomeasure, OsteoMetrics). Alcian blue staining was performed to examine cartilage tissue. Femoral trabecular bone parameters were calculated in an area starting 0.5 mm proximal to the distal growth plate and extending 1 mm (cortical bone excluded). A minimum of three femoral sections was analyzed per animal.

### Micro-CT X-ray tomography

The distal femur or proximal tibia from age and sex-matched mice were scanned with the Skyscan 1174 compact micro-CT system (Bruker-microCT, Aartselaar, Belgium) at a pixel size of 6.03 µm. Datasets were reconstructed using modified cone beam reconstruction software (NRecon) based on the Feldkamp algorithm and segmented into binary images using adaptive local thresholding. Bone volume analysis was performed using the CTan software (Bruker-microCT). Femoral or tibial trabecular bone analysis was performed in a region of interest within the secondary spongiosa starting 0.5 mm from the growth plate and extending 1 mm in height. Mid-diaphysis cortical volume was assessed in a region 4 mm from the growth plate and extending 1 mm in height. Three dimensional surface-rendered models were generated using CTan software (Bruker-microCT) and visualised using CTVol (Bruker-microCT).

### Cell cultures

Bone marrow monocytes (BMM) were collected from the hindlimbs of age and sex-matched PKC-δ KO and WT mice. BMM were cultured in α-MEM (10% FCS, Pen-Strep, GlutaMax) and 1/20 dilution of murine M-CSF conditioned medium [23]. Osteoclast formation from BMM was induced by addition of 100 ng/ml of Glutathione S-transferase (GST)-RANKL [24]. Giant Cell Tumor of bone was cultured as previously described [25]. Osteoclast bone resorption assays were performed using RANKL-induced osteoclasts cultured on Biocoat collagen-1 coated 6-well plates (Becton Dickinson). Mature osteoclasts were seeded onto bovine cortical bone slices at a density of 6×10^3^/well in a 96-well plate for 48 hours. Cells were fixed with 4% paraformaldehyde and osteoclasts visualized by staining for TRAP. Resorption was imaged using Scanning Electron Microscopy (SEM). Pit depth measurements were performed using reflected light microscopy. Carboxy-terminal collagen crosslinks (CTX) in medium were determined using CrossLaps for Culture ELISA kit (Immunodiagnostic Systems, Scottsdale, AZ, USA) according to the manufacturer's instruction. Primary calvarial osteoblasts were prepared from the calvaria of neonatal C57BL/6 mice by enzymatic digestion using Collagenase Type 2 [26]. To prepare co-cultures, calvarial osteoblasts were seeded onto a 96-well plate at 5×10^3^ cells/well in complete α-MEM with 10 nM 1α,25-Dihydroxyvitamin D3 (Sigma-Aldrich). BMMs were seeded with osteoblast cultures at 1×10^4^/well. Co-cultures were treated with 10 nM 1α,25-Dihydroxyvitamin D3 for seven days or until osteoclasts formed.

### Flow cytometry

The BD Biosciences protocol was used to immunostain mouse bone marrow cells. In brief, bone marrow cells were extracted from mice hindlimbs. Red blood cells (RBCs) were lysed in ammonium chloride lysis buffer (0.15 M NH_4_Cl, 10 mM Tris-HCl, 0.1 mM EDTA) and the cells were resuspended in ice cold wash buffer (1% FBS, 0.1% NaN_3_ in PBS) at a concentration of 2×10^7^/ml. Bone marrow cell suspension (10^6^ cells) was incubated with CD45R-FITC, CD3-FITC and CD11b-PE antibodies for 30 minutes in the dark. The cells were washed twice (∼10^6^ cells) and transferred to flow cytometer tubes containing cold wash buffer with 7-AAD (BD Biosciences). The FACSCalibur flow cytometer (BD Biosciences) was used for analysis. Dead cells stained positive for 7-AAD were excluded.

### MTS cell proliferation assay

The Promega CellTiter 96® AQueous MTS cell proliferation assay (Cat#G5421) was used according to the manufacturer's protocol (Promega). Absorbance at 490 nm was quantified using a spectrophotometer.

### Immunofluorescence

Osteoclasts cultured on coverslips or bone discs were fixed and permeabilized with 0.1% Triton X-100. Cells were washed twice with 0.2% BSA in PBS and incubated with anti-mouse α-tubulin (Sigma) and rhodamine-conjugated phalloidin 546 antibody (Molecular Probes). Alpha-tubulin was detected using a goat anti-mouse Alexa Fluor 488 secondary antibody. pMARCKS was detected using a goat anti-rabbit Alexa Fluor 488 secondary antibody. Nuclei were stained with Hoechst 33258 or DAPI nucleic acid stain (Molecular Probes). Cells were imaged with a Nikon A1Si confocal microscope with 405, 488 and 561 nm lasers utilizing a Nikon Apo VC 20× NA 0.75 lens [27].

### Acridine Orange acidification assay

The Acridine Orange assay was performed using age and sex-matched WT and PKC-δ KO BMMs and osteoclasts. Cells were incubated with 10 μg/ml of Acridine Orange (Sigma) for one hour at 37°C, 5% CO_2_, 95% air. Cells were washed in PBS and stored in HANKS buffer. Green fluorescence intensity was measured using a PolarStar Optima fluorescent microplate reader (BMG) with a 485 nm excitation filter and a 520 nm emission filter [28].

### RT-PCR

Total RNA extraction from cells was done using the RNeasy® Mini Kit (QIAGEN) according to manufacturer instructions. For RT-PCR, single-stranded cDNA was reverse transcribed from total RNA using reverse transcriptase with oligo-dT primer. All PCR was carried out using the cycling conditions: 94°C, 40 secs; 58–60°C, 40 secs; and 72°C, 50 secs for 25–30 cycles. The following primers were used: CTR (Forward, 5′- TGGTTGAGGTTGTGCCCA -3′; reverse, 5′- CTCGTGGGTTTGCCTCATC -3′), CsK (Forward, 5′- GGGAGAAAAACCTGAAGC -3′; reverse, 5′- ATTCTGGGGACTCAGAGC -3′), DC-STAMP (Forward, 5′- CTTGCAACCTAAGGGCAAAG -3′; reverse, 5′- TCAACAGCTCTGTCGTGACC -3′), PKC-α (Forward, 5′- GAAGGTGATGCTTGCTGACA -3′; reverse, 5′- CGTTGACGTATTCCATGACG -3′), PKC-β (Forward, 5′- TCCCTGATCCCAAAAGTGAG -3′; reverse, 5′- AACTTGAACCAGCCATCCAC -3′), PKC-γ (Forward, 5′- GCAGCTTCACTCCACCTTTC -3′; reverse, 5′- CCTGTAGATGATGCCCTGGT -3′), PKC-ε (Forward, 5′- GAGGACTGGATTGACCTGGA -3′; reverse, 5′- ATCTCTGCAGTGGGAGCAGT -3′), PKC-η (Forward, 5′- CATCCCACACAAGTTCAACG -3′; reverse, 5′- ATATTTCCGGGTTGGAGACC -3′), PKC-θ (Forward, 5′- CCAGAAAAAGCCAACCATGT -3′; reverse, 5′- GGAACATGGTTTCTCGGCTA -3′), PKC-ζ (Forward, 5′- AAGTGGGTGGACAGTGAAGG -3′; reverse, 5′- TCGTGGACAAGCTCCTTCTT -3′), PKC-ι/λ (Forward, 5′- TATGGCTTCAGCGTTGACTG -3′; reverse, 5′- CCTTTGGGTCCTTGTTGAGA -3′), TRAP (Forward, 5′- TGTGGCCATCTTTATGCT -3′; reverse, 5′- GTCATTTCTTTGGGGCTT -3′), 36B4 (Forward, 5′- TCATTGTGGGAGCAGACA -3′; reverse, 5′- TCCTCCGACTCTTCCTTT -3′). PCR samples were analyzed by DNA agarose gel electrophoresis.

### cDNA Microarray Array

Microarray analysis of PKC family gene expression during osteoclast differentiation was performed according to the manufacturer's instructions (Research Genetics). BMM cells were treated with 100 ng/ml RANKL for 5 days to differentiate into mature osteoclasts. Total RNA was isolated from the cultures at the time points using a commercially available RNA extraction kit (Qiagen, Victoria, Australia), according to the manufacturer's instructions. The RNA concentration was determined by measuring the absorbance at 260 nm with a Nano-drop 2000 (Thermo Scientific). Gene expression of PKC family members during in vitro osteoclast differentiation was detected by cDNA microarray.

### Western Blot Analysis

Total cellular proteins were extracted from cultured cells using RIPA lysis buffer (50 mM Tris pH7.5, 150 mM NaCl, 1% v/v Nonidet P-40, 0.1% SDS, 1% sodium deoxycholate) supplemented with Protease Inhibitor Cocktail (Roche), 50 µg/ml PMSF and 1 mM Na_3_VO_4_. Lysates were cleared by centrifugation at 16,000 g for 10 mins at 4°C and supernatant was collected. For western blotting, extracted proteins diluted in SDS-sampling buffer were resolved by SDS-PAGE (10%) gels and then electroblotted onto Hybond-C nitrocellulose membranes (Amersham Life Science). Following transfer, membranes were blocked with 5% w/v skim milk in TBS-Tween (TBS; 0.05 M Tris, 0.15 M NaCl, pH 7.5 and 0.2% v/v Tween-20) for 1 hour and then probed with primary antibodies diluted in 1% w/v skim milk powder in TBS-Tween for 2 hours. Membranes were washed in TBS-Tween and then incubated with HRP-conjugated secondary antibodies. Antibody reactivity was detected using the Western Lightning Ultra chemiluminescence substrate (Perkin-Elmer) according to manufacturer's instructions. The membrane was developed using the FujiFilm LAS-3000 Gel Documentation System (FujiFilm).

### Statistical Analysis

Single comparison tests were done by two-tailed t-test using STATA software (Statacorp). The results are representative of at least three independent experiments. For comparisons between multiple means, a one-way ANOVA (Bonferroni post-hoc test) was used. P-values<0.05 were considered significant. All charts and data are represented as mean ± standard deviation (SD).

## Results

### PKC-δ is a predominant isoform among all PKC family genes expressed in osteoclasts and plays an important role in bone resorption in vitro

**Figure 1:**
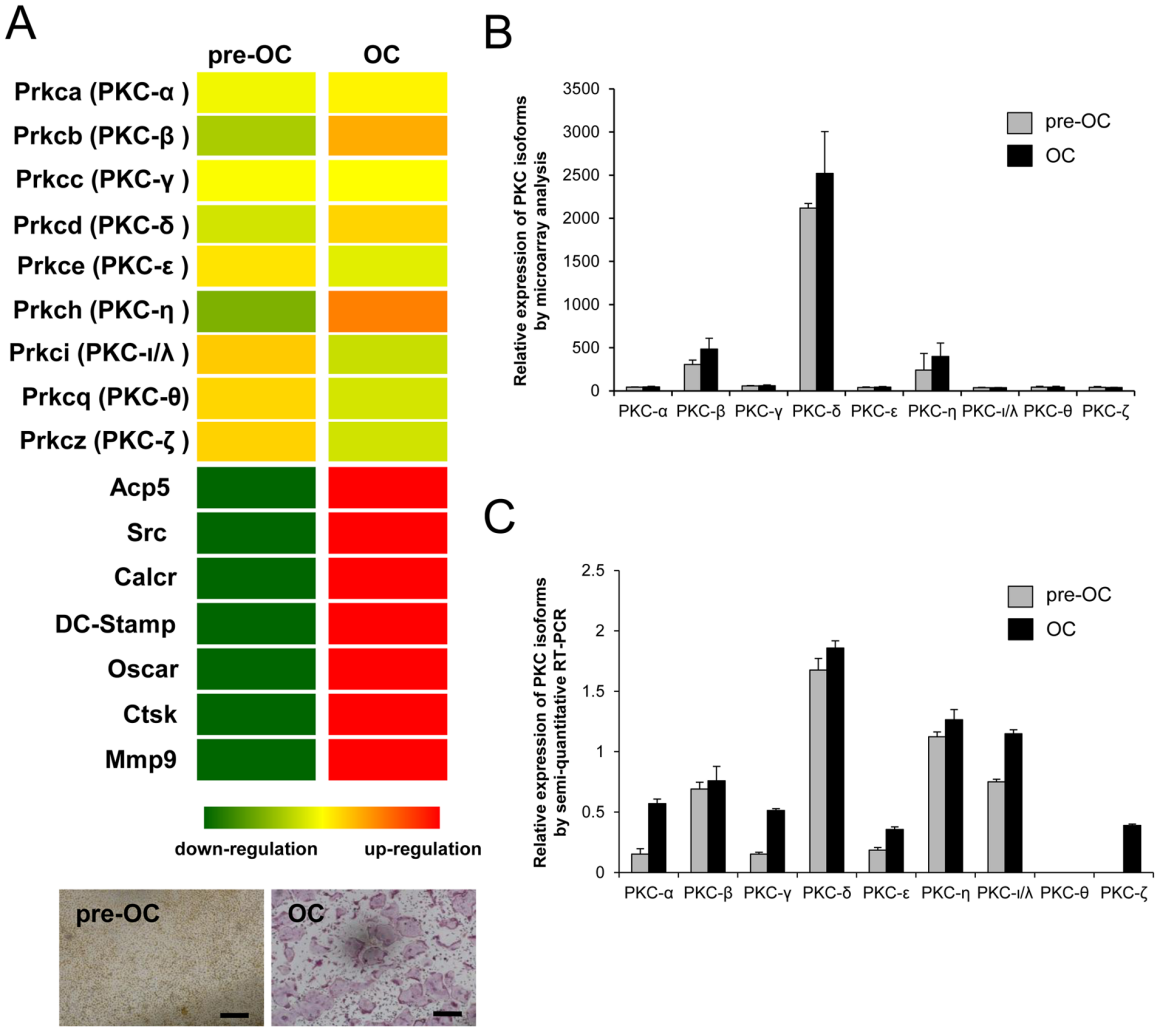
PKC-δ is the predominant isoform among PKCs expressed in osteoclasts. Microarray analysis of PKC isoform gene expression during osteoclast differentiation. (A) BMM cells (pre-OC) were treated with 100 ng/ml RANKL for 5 days to differentiate into mature osteoclasts (OC). Total RNA was harvested for microarray analysis. Heatmap demonstrating the upregulation of PKC-β, PKC-δ and PKC-η during osteoclast differentiation, with osteoclast specific genes. Up-regulation and down-regulation are shown in red and green respectively. TRAP staining for osteoclasts was also included in parallel experiment. Scale bar represents 200 μm. (B) Relative expressions of PKC isoforms was presented by arbitrary readings of microarray analysis (C) Semi-quantitative RT-PCR analysis comparing the gene expression profile of PKC isoforms in BMM and RANKL-treated osteoclasts including classical PKCs (−α, −β and −γ), novel PKCs (−δ, −ε, −η and −θ) and atypical PKCs (−ι/λ and −ζ).

To examine the involvement of individual PKC isoforms in the regulation of bone homeostasis and osteoclast signaling, we compared the gene expression profile of all the PKC family of genes expressed in osteoclasts using microarray analysis. The results show that PKC-δ is most abundantly expressed in osteoclasts among the PKC family of genes (Fig. 1A–B), which includes classical PKCs (−α, −β and −γ), novel PKCs (−δ, −ε, −η and −θ) and atypical PKCs (−ι/λ and −ζ). The gene expressions of PKC isoforms during osteoclast differentiation were further examined by semi-quantitative RT-PCR (Fig. 1C). The result confirmed that PKC-δ is most abundantly expressed among the PKC isoforms.

**Figure 2:**
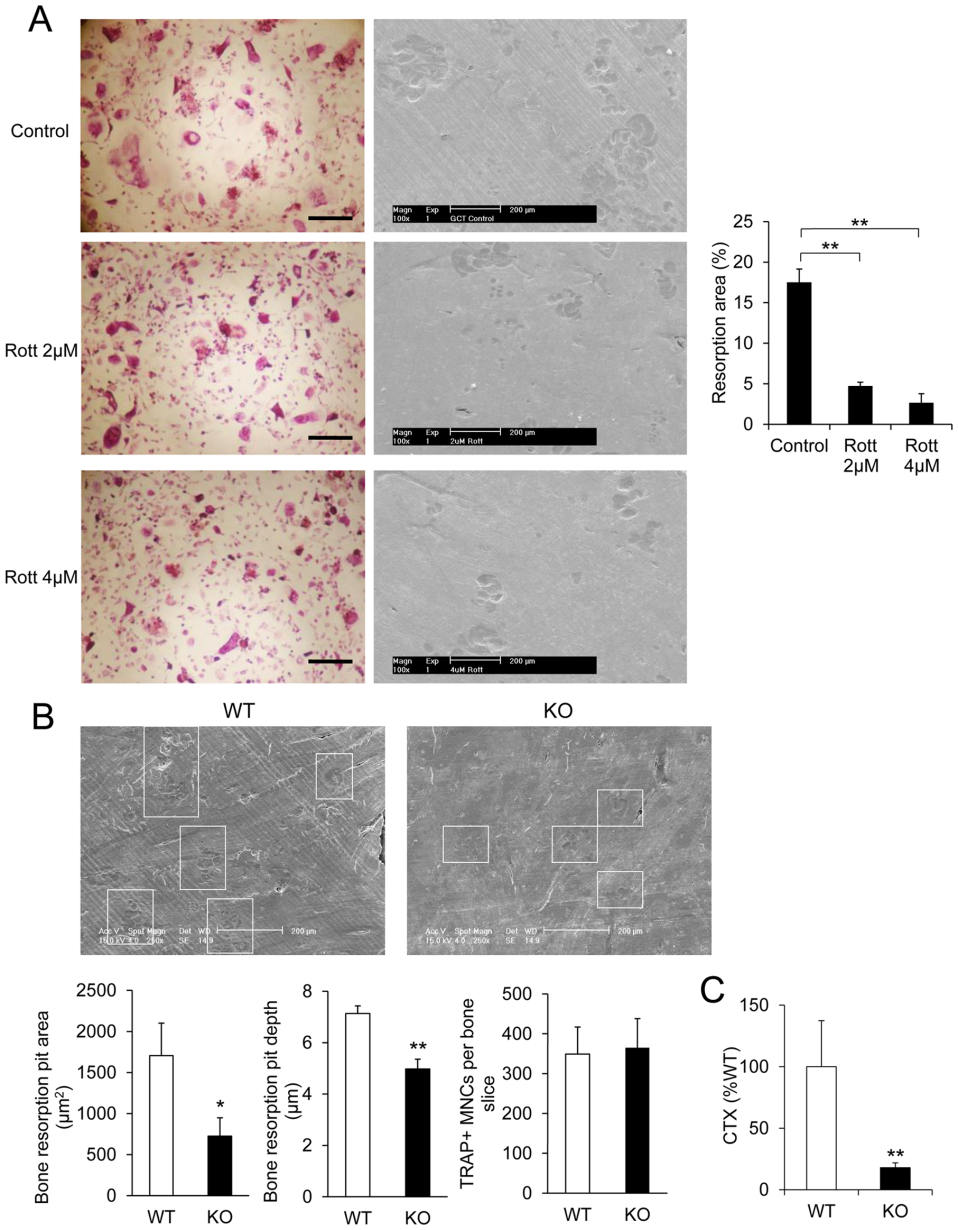
Inhibition of PKC-δ and knock out of PKC-δ resulted in impaired osteoclastic bone resorption in vitro. (A) Multinucleated giant cells isolated from patients presenting with Giant cell tumor (GCT) of bone were cultured on the bovine bone slices in the presence and absence of Rottlerin (Rott). Representative light images of osteoclasts derived from GCT, and scanning electron micrographs of resorptive lacunae on bone slices. Resorbed area as a percentage of total bone slice area was determined. (B) SEM micrographs of bone discs cultured with WT and PKC**-**δ KO osteoclasts. Osteoclast bone resorption pits are highlighted by white boxes. Average bone resorption area and average pit depth was measured. Total osteoclast numbers were the same on all bone discs. (C) Percentage of CTX released into culture medium by PKC-δ KO and WT osteoclasts cultured in bone. Scale bar represents 200 μm. Bar charts represent mean ± standard deviation. *, p-value <0.05, **, p-value <0.01.

Next, we conducted two sets of experiments to determine the role of PKC-δ in osteoclast function (Fig. 2). We found that inhibition of PKC-δ by Rottlerin (Fig. 2A) or KO of PKC-δ in mice (Fig. 2B) resulted in impaired osteoclastic bone resorption. Both the average bone resorption pit area and bone resorption pit depth were significantly lower in KO osteoclasts as compared to WT osteoclasts (Fig. 2B). Moreover, levels of carboxy-terminal collagen crosslinks (CTX) were measured in medium from osteoclasts cultured on bone, and revealed a significant decrease in PKC-δ KO osteoclasts (Fig. 2C). We conclude that PKC-δ is the predominant isoform among the PKC family of genes that is expressed in osteoclasts and plays an important role in bone resorption.

### Genetic deletion or inhibition of PKC-δ protects against LPS-induced osteolysis in vivo

**Figure 3:**
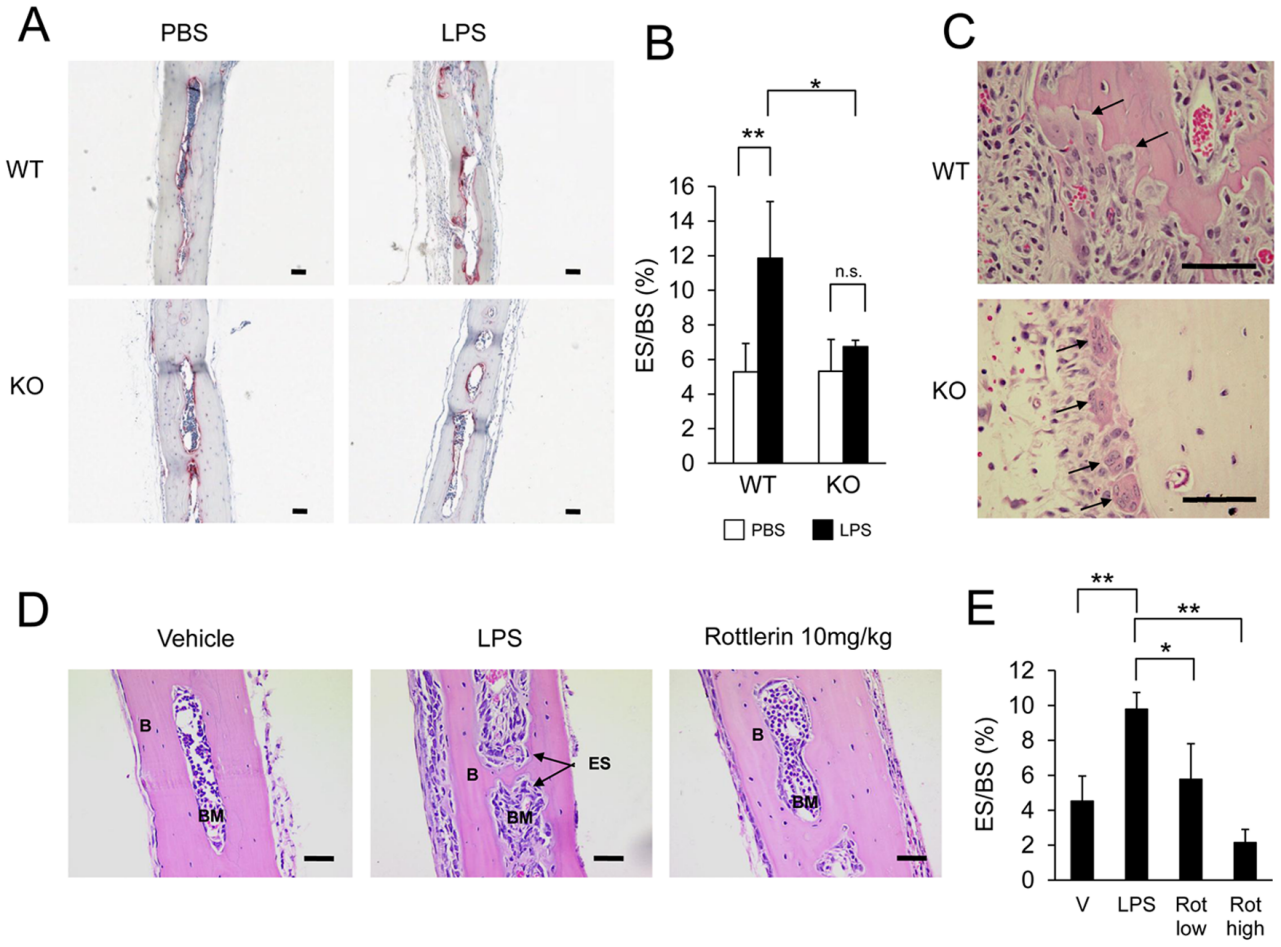
PKC-δ deficiency protects against LPS-induced osteolysis. (A) Representative TRAP stained histological sections of calvarial bone from WT and PKC-δ KO mice seven days post-injection with Phosphate Buffered Saline (PBS) or Lipopolysaccharide (LPS). (B) Bone eroded surface quantified by bone histomorphometry (n = 4). (C) H&E stained sections of LPS-treated calvaria. Active bone resorbing WT osteoclasts and unattached inactive KO osteoclasts are indicated by arrows. (D) H&E stained sections of WT mice (n = 4) seven days post injection with either Vehicle (V), Lipopolysaccharide (LPS), LPS with 2 mg/kg Rottlerin (Rot low) or LPS with 10 mg/kg Rottlerin (Rot high). (E) Bone eroded surface of Rottlerin treated bone quantified by bone histomorphometry. Unshaded bars in bar charts denote vehicle injections, shaded bars denote LPS injections. Bar charts represent mean ± standard deviation. Scale bar represents 100 μm. *, p-value <0.05. **, p-value<0.01, n.s., no significance (p-value>0.05).

To further explore the impact of PKC-δ-deficiency in vivo under pathological settings, we next examined the capacity of PKC-δ to protect against LPS-induced osteolysis. To this end, subcutaneous injections of PBS or LPS were administered to the calvaria of WT and PKC-δ KO mice. Mice were sacrificed seven days post injection and the calvaria were dissected for histological analysis (Fig. 3A). Bone histomorphometry revealed no differences in the eroded surface of the calvaria of LPS-injected PKC-δ KO mice compared to PBS controls. In contrast, significant bone erosion was observed in calvaria of LPS-treated WT mice (Fig. 3B). Consistent with the lack of LPS-induced bone erosion, a number of osteoclasts were observed actively resorbing the bone surface in WT calvaria samples but in contrast, KO osteoclasts appeared to be largely unpolarized, lacking ruffled borders and in proximity, but not attached to the bone surface (Fig. 3C). This data is consistent with in vitro data suggesting that osteoclasts derived from PKC-δ-deficient mice are dysfunctional. Moreover, these findings indicate that inhibition of PKC-δ activity protects against pathological osteolysis in vivo.

To complement these findings, we also investigated the potential therapeutic effects of PKC-δ inhibition on osteolysis by assessing the effects of the PKC-δ inhibitor Rottlerin on LPS-induced bone loss. For this purpose, subcutaneous injections with LPS, LPS and 2 mg/kg of Rottlerin, LPS and 10 mg/kg of Rottlerin, or the vehicle (PBS) were administered to the calvaria of age and sex-matched WT mice. Seven days post injection, the calvaria were dissected out for histological analysis (Fig. 3D). Bone histomorphometry revealed a significant increase in osteoclastic bone erosion in the LPS-positive control (Fig. 3E). Interestingly, LPS injection with 2 mg/kg and 10 mg/kg of Rottlerin resulted in a significant dose-dependent decrease in the eroded surface compared to the LPS group (Fig. 3E). Taken together, loss of PKC-δ protects against LPS-induced osteolysis, and treatment with the PKC-δ inhibitor Rottlerin blocks LPS-induced bone resorption in mice.

### PKC-δ-deficient mice exhibit increased bone mass with features of osteoclast defective osteopetrosis

**Figure 4:**
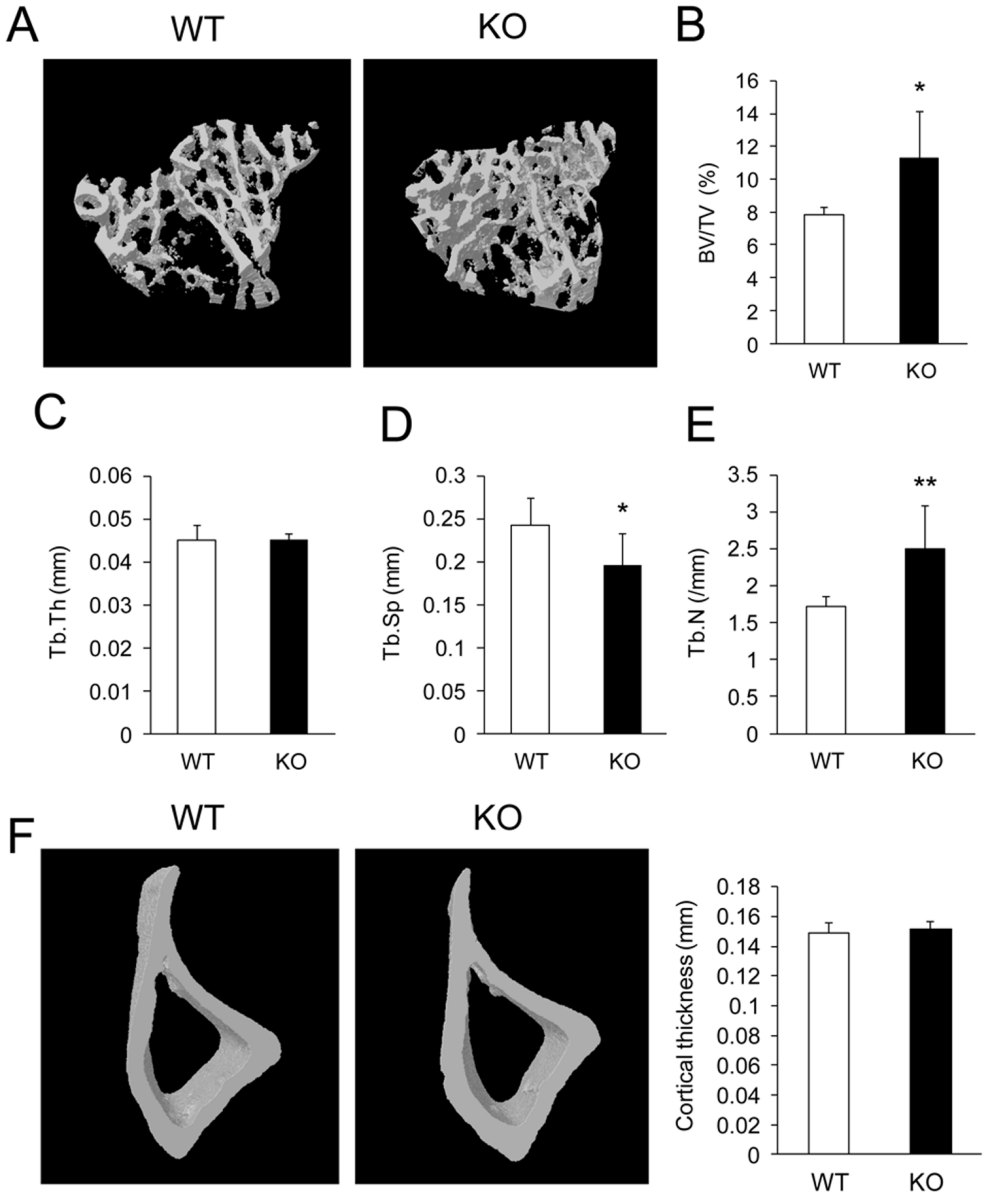
PKC-δ deficiency increases trabecular bone volume. (A) Micro-CT images of the trabecular bone in the proximal tibial metaphysis of three-month-old female PKC-δ KO mice and age-sex matched WT mice. (B–E) Micro-CT analysis of eight pairs of three-month-old female PKC-δ KO and WT mice tibias for trabecular bone volume (BV/TV), trabecular thickness (Tb.Th), trabecular separation (Tb.Sp) and trabecular number (Tb.N). (F) Micro-CT analysis of tibial cortical thickness measured 5 mm distal to the proximal growth plate. Bar charts represent mean ± standard deviation. *, p-value <0.05. **, p-value<0.01.

To address the role of PKC-δ in bone homeostasis we directly assessed the bone phenotype of PKC-δ deficient mice by micro-CT (Fig. 4A). Analysis of trabecular bone parameters revealed a statistically significant increase in trabecular bone volume in PKC-δ KO mice by approximately 45% over WT mice (Fig. 4A and B). This increase was accompanied by reduced trabecular separation (Tb.Sp) (Fig. 4D) and increased trabecular number (Tb.N) (Fig. 4E) in PKC-δ KO mice. No differences were observed in trabecular thickness (Tb.Th) and cortical thickness (Fig. 4C and F). Collectively, these data indicate that PKC-δ KO mice exhibit a high bone mass phenotype consistent with osteopetrosis due to defective osteoclast function.

**Figure 5:**
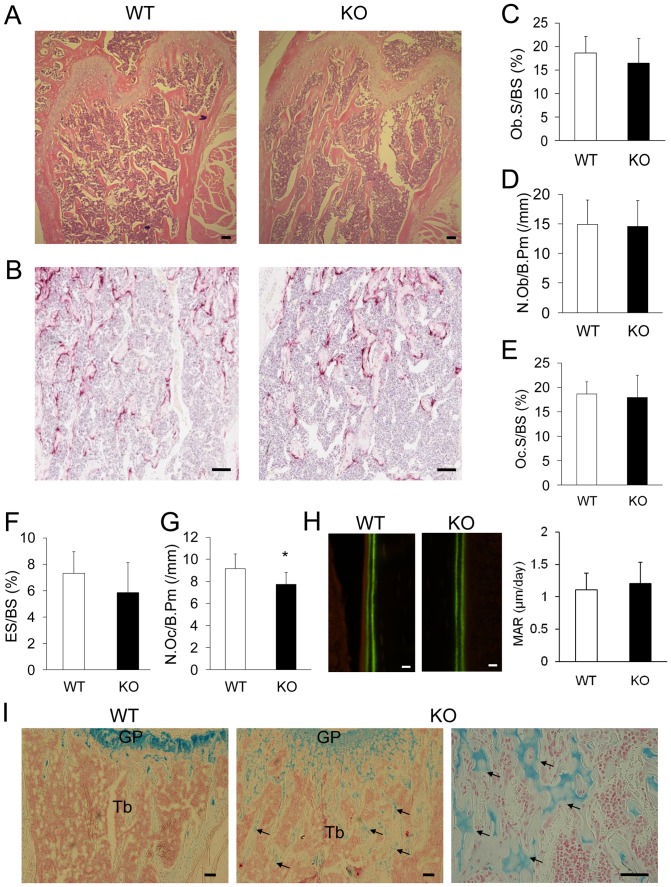
Osteoclast deficiencies in trabecular bone of PKC-δ KO mice. (A–B) Histological sections of three-month-old female PKC-δ KO mice and age-sex matched WT mice femurs stained with H&E (A) and TRAP (B), scale bar represents 100 µm. (C–G) Osteoblast surface (Ob.S/BS), number of osteoblasts (N.Ob/B.Pm), osteoclast surface (Oc.S/BS), eroded surface (ES/BS) and number of osteoclasts (N.Oc/B.Pm) was analyzed for eight pairs (n = 8) of three-month-old, female PKC-δ KO and WT mice femurs. (H) Bone mineral apposition rate measured from calcein-labeled bones, scale bar represents 10 µm. (I) Alcian Blue stained histological sections; far right image shows a higher magnification micrograph of unresorbed cartilage in PKC-δ KO bone, scale bar represents 100 µm. GP; growth plate, Tb; Trabecular bone. Arrows indicate the remnants of unremodeled cartilage matrix within trabecular bone. Bar charts represent mean ± standard deviation. *, p-value <0.05. **, p-value<0.01.

To further identify specific cellular changes (osteoblast and osteoclast number) at the trabecular bone surface of PKC-δ-deficient mice, bone histomorphometry was performed. To this end, H&E stained and TRAP stained histological sections of femurs from three month old female WT and PKC-δ KO mice were used for histomorphometric analysis (Fig. 5A and B). As illustrated in Figure 5C, there were no significant differences observed in osteoblast surface (Ob.S/BS) and number of osteoblasts (N.Ob/B.Pm) (Fig. 5D) in PKC-δ KO mice as compared to WT controls. Analysis of TRAP stained histological sections identified a marginal reduction in osteoclast number (N.Oc/B.Pm) (Fig. 5G) in PKC-δ KO bones but there were no observable differences in either the osteoclast surface (Oc.S/BS) and eroded surface (ES/BS) (Fig. 5E and F). Together, these histological data imply an increase in trabecular bone volume in PKC-δ-deficient mice.

Previously it has been shown that PKC-δ KO mice exhibit a defect in embryonic bone formation [21]. In comparison, in adult mice we did not detect a significant difference in mineral apposition rate in PKC-δ KO mice when compared to WT controls (Fig. 5H). Furthermore, alcian blue staining revealed remnants of unresorbed cartilage embedded in the centre of mineralized osseous tissue of PKC-δ KO bones (Fig. 5I). The presence of unresorbed cartilage within the trabecular bone suggests impaired resorption of growth plate cartilage and is one of the characteristic pathological features of osteopetrosis [29]–[31]. This data further supports the notion that PKC-δ-deficient mice display mild osteopetrosis predominantly owing to an osteoclast defect.

### Enhanced osteoclastogenesis in PKC-δ KO bone marrow monocytes

**Figure 6:**
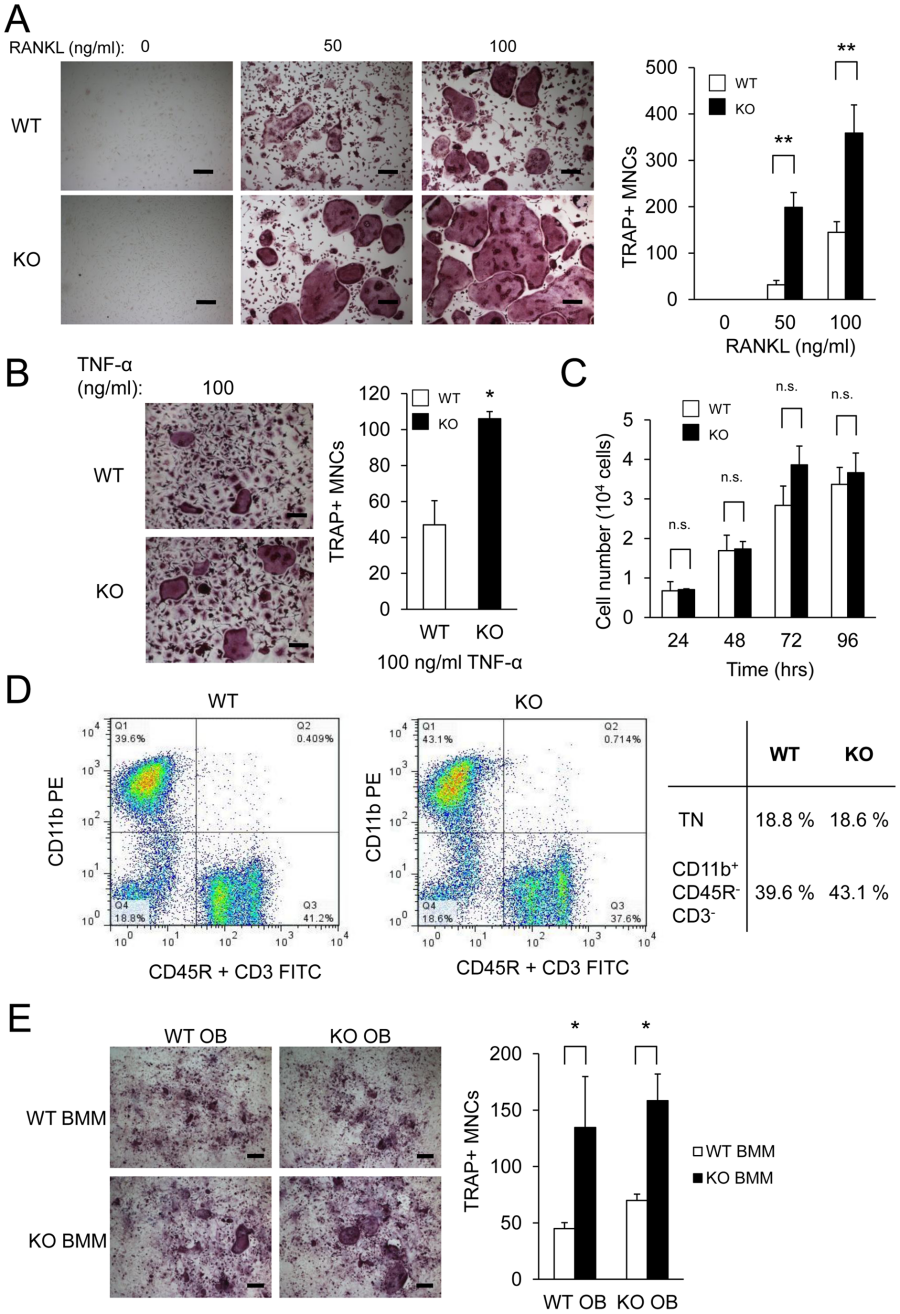
Altered RANKL-induced osteoclastogenesis in PKC-δ KO mice. (A) Bone marrow monocytes (BMMs) from age-sex matched PKC-δ KO and WT mice hindlimbs were stimulated with M-CSF and different concentrations of RANKL (0, 50 ng/ml and 100 ng/ml) for four days to form osteoclasts. The cells were fixed and TRAP stained to quantify the number of multinucleated osteoclasts (>3 nuclei). Experiments were performed in triplicate. (B) WT and KO BMM stimulated with MCSF and 100 ng/ml of TNF-α to form TRAP positive osteoclasts. Scale bar represents 200 μm. Bar charts represent mean ± standard deviation. (C) MTS cell proliferation assay was performed on M-CSF treated cells stimulated with RANKL at the indicated times. Scale bar represents 200 μm. (D) Bone marrow cells from WT and PKC-δ KO mice were immunostained for CD45R, CD3 and CD11b for flow cytometry analysis and the osteoclast precursor population in WT and PKC-δ KO bone marrow was quantified (TN, triple negative). Charts are presented as pseudocolour density plots. (E) TRAP-stained primary osteoblast and BMM cocultures from WT and PKC-δ KO mice stimulated with 10 nM of Vitamin D3 for seven days. Scale bar represents 200 μm. Bar charts represent mean ± standard deviation. *, p-value <0.05. **, p-value<0.01.

To investigate the effects of PKC-δ deficiency on osteoclast formation, osteoclasts were cultured from BMMs from PKC-δ KO mice. As shown in Figure 6A, PKC-δ KO BMM produced significantly more osteoclasts than WT at all RANKL doses. Similarly, TNF-α stimulation induced formation of TRAP-positive multinucleated osteoclasts with KO BMMs forming significantly more osteoclasts than WT BMMs (Fig. 6B). Previous PKC-δ KO mice studies had identified changes in cell proliferation in vascular and lymphoid systems [22], [32]. To determine if proliferation rates were altered in PKC-δ KO BMMs relative to WT cells an MTS proliferation assay was performed. However, there were no significant differences in M-CSF-induced cell proliferation between WT and KO BMMs (Fig. 6C).

Increased osteoclast numbers in vitro could be accounted for by changes in the proportion of osteoclast progenitor cells in the bone marrow of PKC-δ KO mice. It has been previously identified that an osteoclast progenitor cell population lies within the CD45R^−^CD3^−^CD11b^low/−^ bone marrow population [33]. Flow cytometry was used to assess differences in the population of osteoclast progenitor cells in PKC-δ KO mouse bone marrow. The CD45R^−^CD3^−^CD11b^low/−^ [33] fraction in KO bone marrow was similar to WT (Fig. 6D). This suggests that the increased osteoclast formation was not caused by differences in the osteoclast progenitor cell population within the triple negative fraction of KO bone marrow.

In a physiological setting, osteoblasts express RANKL, M-CSF, and OPG to regulate osteoclasts in a paracrine manner [4], [34], [35]. In order to test if PKC-δ KO osteoblasts can support osteoclastogenesis, an osteoblast-BMM co-culture assay was performed. As shown in Figure 6E, co-cultures with KO BMMs produced significantly more osteoclasts than from WT BMMs regardless of the osteoblast genotype employed, further supporting an autonomous osteoclast defect. Taken together, these results suggest that PKC-δ KO mice exhibit altered osteoclastogenesis in an attempt to compensate for an intrinsic osteoclast bone resorption defect.

### Altered gene expression profile of Cathepsin K, and PKC-α, PKC-γ and PKC-ε in osteoclasts derived from PKC-δ KO mice

**Figure 7:**
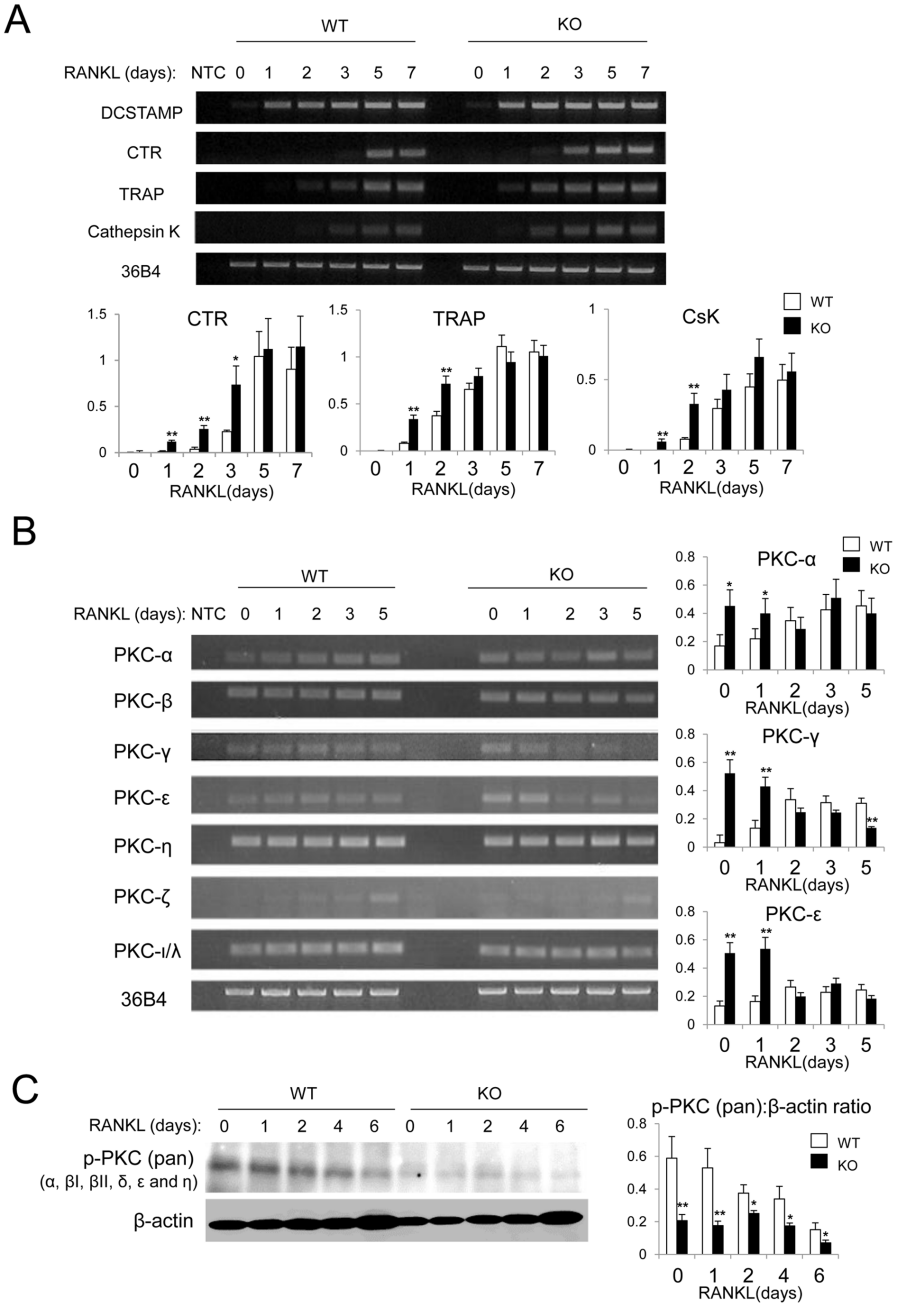
Altered gene expression profile in PKC-δ KO osteoclast cultures. BMMs from WT and PKC-δ KO mice were stimulated with 100 ng/ml of RANKL for the indicated times (0, 1, 2, 3, 5 and 7 days). Total RNA was extracted for RT-PCR. (A) Gene expression of osteoclast specific genes: DC-STAMP, Calcitonin receptor (CTR), Tartrate-resistant acid phosphatase (TRAP), Cathepsin K (CsK) and reference gene 36B4. Quantitative gene expression relative to 36B4 as determined by densitometry of agarose gel images. NTC, no template control. (B) PKC-δ KO BMM showed altered gene expression of PKC isoforms during RANKL-induced osteoclastogenesis. Quantitative gene expression relative to 36B4 of PKC-α, PKC-γ and PKC-ε by densitometry of agarose gel images. (C) Western blot analysis showing that the phosphorylation levels of PKC isoforms were reduced in PKC-δ KO osteoclasts compared to WT. Statistical analysis was performed by comparing to WT in each time point. *, p-value <0.05. **, p-value <0.01.

To examine the gene expression profile of osteoclasts in PKC-δ KO cells, a RANKL time course was performed on BMM cells from age and sex-matched WT and PKC-δ KO bone marrow. Total RNA was extracted and RT-PCR was performed (Fig. 7). The osteoclast marker genes calcitonin receptor (CTR), TRAP and Cathepsin K (CsK) were increased during the early stages of RANKL stimulation (Days 1–3) in KO BMMs compared to WT (Fig. 7A).

The functions of PKC family genes may be interdependent and co-regulated, thus, the gene expression of the other PKC isoforms was investigated during RANKL-induced osteoclastogenesis. PKC-α, PKC-γ and PKC-ε gene expression appears to be increased in KO BMM up to day 1 of RANKL stimulation and returns to WT levels during osteoclastogenesis (Fig. 7B). Interestingly, western blot analysis demonstrated that the phosphorylation levels of PKC isoforms were decreased in PKC-δ KO osteoclasts (Fig. 7C), indicating the overall PKC activities were reduced. Collectively, these results suggest that PKC-δ KO osteoclasts have an altered gene expression profile in an apparent attempt to compensate for an intrinsic osteoclast defect.

### Altered osteoclast signaling pathways of pERK and pcSrc in PKC-δ KO cells

**Figure 8:**
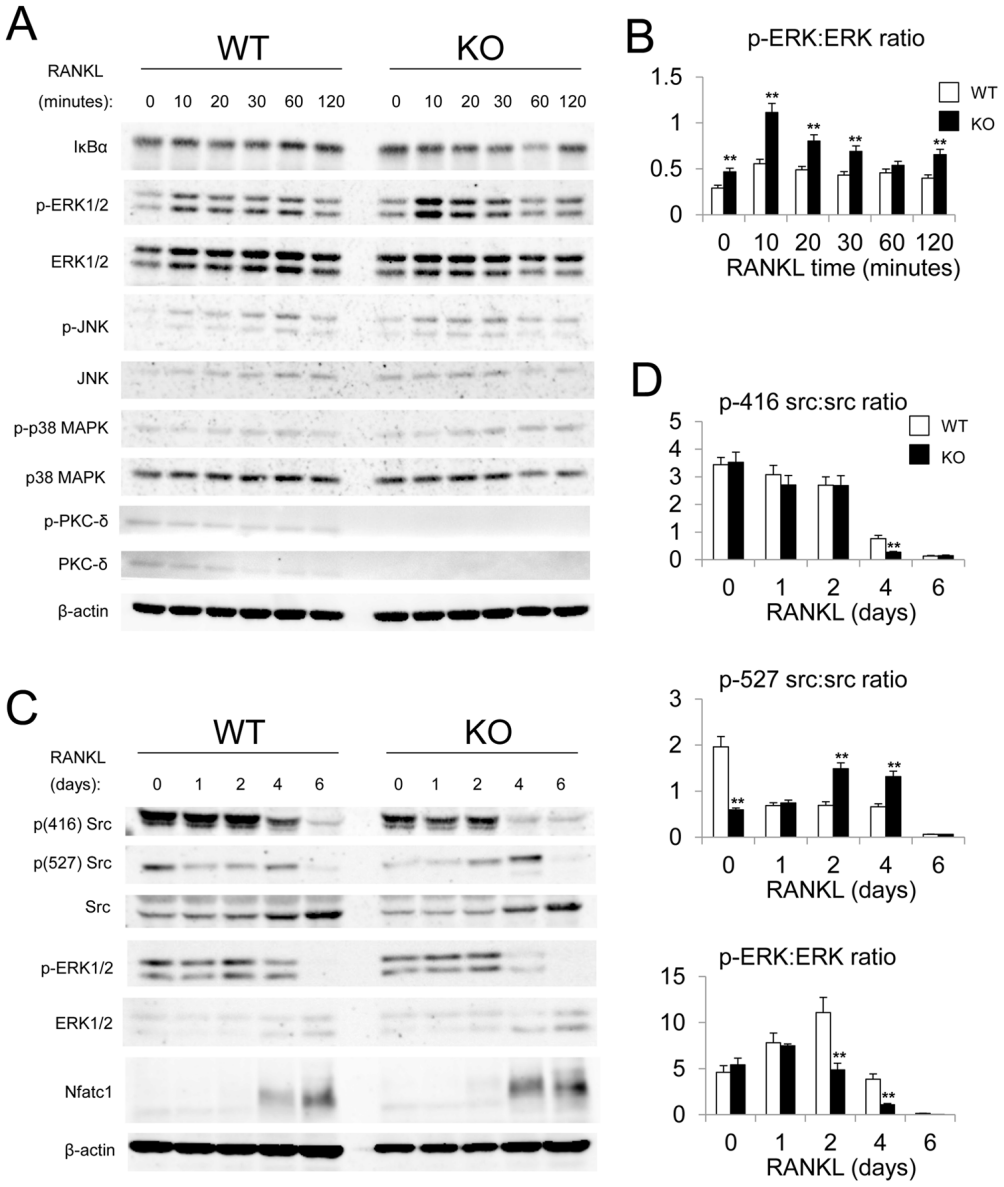
Altered ERK and Src signaling in PKC-δ deficient osteoclasts. WT and PKC-δ KO BMMs were serum-starved overnight before stimulation with M-CSF and 100 ng/ml of RANKL at the indicated times. (A) Western blot analysis of total protein from WT and PKC-δ KO BMMs stimulated with M-CSF and 100 ng/ml of RANKL (short time-course, 0–120 min). (B) Quantitative analysis of short-term ERK phosphorylation relative to total ERK protein expression by densitometry of western blot images. (C) Western blot analysis of RANKL-induced osteoclastogenesis (long time-course, 0–6 days). (D) Quantitative analysis of Src Tyr-416 and Tyr-527 phosphorylation status relative to total Src protein expression, and long-term ERK phosphorylation relative to total ERK protein expression, as measured by densitometry of western blot images. β-actin was probed as a loading control. Statistical analysis was performed by comparing to WT in each time point. *, p-value <0.05. **, p-value<0.01.

To investigate the potential signaling cascade through which PKC-δ modulates osteoclastogenesis, we next compared the activation of prototypical RANKL-signaling pathways between BMMs derived from PKC-δ KO and WT mice by western blot. For this purpose, a short term (0, 10, 20, 30, 60, and 120 mins) and a long term (0, 1, 2, 4, and 6 days) RANKL-induced osteoclastogenesis time course was performed on WT and KO cells and major osteoclast signaling pathways, IκBα (NF-κB signaling), ERK, JNK, p38 MAPK, Src, and NFATc1 examined. As shown in Figure 8A–B, the protein expression and/or phosphorylation levels of IκBα, JNK and p38 MAPK were comparable between WT and KO cells within two hours of RANKL stimulation. By comparison, phosphorylated ERK1/2 levels were increased up to 2-fold higher than in the WT at 10 minutes of RANKL stimulation (Fig. 8B), indicative of enhanced osteoclast differentiation and survival.

In the longer RANKL-induced osteoclastogenesis time course (six days), PKC-δ KO cells exhibited similar Src protein expression levels to that of the WT. Interestingly, the phosphorylated forms of Tyr-416, in p(416) Src and ERK were reduced in the PKC-δ KO osteoclasts compared to the WT at day four of RANKL stimulation (Fig. 8C–D). By comparison, phosphorylated form of Tyr-527, in p(527) Src was decreased at day 0 and increased at 2 and 4 (Fig. 8C–D). These effects further support the notion that altered signalling pathways in PKC-δ KO osteoclasts lead to enhanced osteoclastogenesis in an attempt to compensate for an intrinsic defect in osteoclast bone resorption.

### The potential role of cytoskeletal reorganization, lysosomal acidification and MARCKS phosphorylation in bone resorption by PKC-δ KO osteoclasts

**Figure 9:**
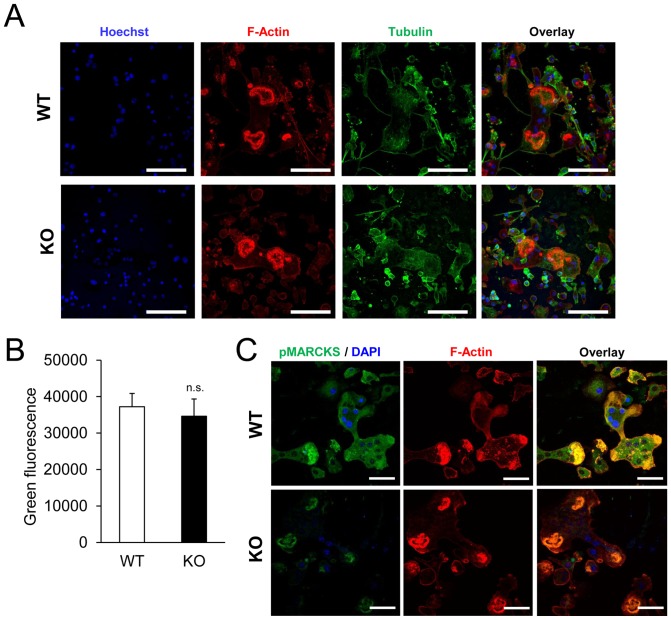
Cytoskeletal reorganization, lysosomal acidification and MARCKS phosphorylation in PKC-δ KO osteoclasts. (A) Osteoclasts on bone slices were immunofluorescently stained with Rhodamine Phalloidin, anti-α-tubulin, and Hoechst to visualize F-actin, α-tubulin and DNA by confocal microscopy. Scale bar represents 100 μm. (B) Osteoclasts were treated with Acridine Orange, which displays green fluorescence at neutral pH. Acridine Orange green fluorescence intensity was measured in a fluorescence microplate reader. Bar charts represent mean ± standard deviation. Experiments were performed in triplicate. n.s., no significance (p-value>0.05). (C) Osteoclasts on bone slices were immunofluorescently stained pMARCKS, Rhodamine Phalloidin and DAPI to examine the phosphorylation levels of MARCKS by confocal microscopy. Scale bar represents 20 μm.

To gain further insight into the underlying cause of the defective bone resorption by PKC-δ KO osteoclasts, the osteoclast cytoskeleton was examined. Both WT and KO osteoclasts exhibit similar cytoskeleton organization as evidenced by the presence of a tight ring of actin at the site of bone attachment called the “F-actin ring” or “sealing zone” (Fig. 9A). Aside from cytoskeletal reorganization and adhesion, lysosome/endosome-mediated acidification of the bone resorption compartment is important for osteoclast function. It was previously shown that PKC-δ inhibition by Rottlerin reduced acidification in bone resorbing osteoclasts [36]. Thus, lysosomal acidification was investigated in PKC-δ KO osteoclasts using the pH-probe, acridine orange, which fluoresces green to orange with increasing intracellular acidification. Acridine orange treated WT and KO osteoclasts show similar levels of green fluorescence (Fig. 9B), indicating that acidification was not affected by loss of PKC-δ. It has been shown that decreased bone resorption in PKC-δ KO osteoclasts is regulated by PKC-δ through the modulation of myristoylated alanine-rich C-kinase substrate (MARCKS) [37], here, we also confirmed that the phosphorylation levels of MARCKS in PKC-δ KO osteoclasts was diminished in the PKC-δ KO osteoclasts by confocal microscopic analysis (Fig. 9C).

## Discussion

PKCs are a family of serine/threonine kinases involved in signal transduction of many cellular processes. Despite the ubiquitous nature of PKCs in cell signaling, studies associating PKCs to osteoclasts remain limited. In the present study we demonstrate that PKC-δ KO mice exhibit a mild osteopetrotic bone phenotype, a function not ascribable to a possible osteoblast defect as previously reported [21]. Moreover we demonstrate that the increased bone mass phenotype is due to a dysfunction in osteoclasts. Specifically, osteoclasts derived from PKC-δ KO mice exhibit impaired bone resorption capacity, owing at least in part to decreased levels of activated p(416) Src.

Previous studies have indicated PKC's have a role in regulating osteoblast and osteoclast activity. In osteoclasts, PKC-α and PKC-β had been shown to regulate osteoclast formation or function in vitro [9], [38]. In osteoblasts, PKC-α and PKC-δ regulate osteoblast proliferation and differentiation respectively [21], [39], [40]. This laboratory and others [36] have implicated PKC-δ in osteoclast biology in vitro. Interestingly, it has been previously reported that PKC-δ-KO mice have a defect in osteoblast differentiation during embryonic development [21]. Here, by examining and characterizing mice lacking PKC-δ, the role of PKC-δ in osteoclastic bone resorption and inflammation-elicited bone loss was further elucidated.

Using micro-CT and histological analysis we have demonstrated that adult PKC-δ-KO mice exhibit increased trabecular bone volume. This bone phenotype was reminiscent of other osteopetrotic gene knockout mice such as PYK2 [41], carbonic anhydrase [42] and Cathepsin K [43] deficient mice, which exhibit a defect in osteoclast function. Bone histomorphometric analysis revealed a reduced number of osteoclasts in PKC-δ KO bones indicating that the bone phenotype was likely attributed to a defect in osteoclasts. This was supported by the presence of cartilaginous remnants in the trabecular bone, a histological hallmark of osteoclast defective osteopetrosis [29]–[31]. Contrary to the previously reported reduction in embryonic bone formation [21], adult PKC-δ KO mice did not appear to have a defect in osteoblast bone formation in vivo. This may indicate dual roles for PKC-δ in regulating bone formation during embryonic bone development and regulating osteoclasts in normal bone homeostasis.

The bone phenotype of PKC-δ KO mice signified a defect in osteoclast function. In contrast, in vitro cultures of BMMs from PKC-δ KO mice showed enhanced osteoclastogenesis, and this was independent of osteoblast activity. This suggests that the enhancement in osteoclastogenesis in PKC-δ-deficient bone marrow was cell autonomous. A possible cause for increased osteoclast formation is alterations in cell proliferation. Indeed, past studies have established a role for PKC-δ in cell proliferation [22], [32], [44]. However, PKC-δ-KO BMMs showed normal cell proliferation. Another possibility is an increased pool of osteoclast progenitors in the bone marrow of PKC-δ-KO mice. Flow cytometry analysis showed the CD11b^low/−^CD45R^−^CD3^−^ fraction in the bone marrow, known to contain an osteoclast progenitor population [33], was similar between WT and KO mice. Interestingly, the pro-survival ERK pathway [45] was upregulated in PKC-δ-KO BMMs undergoing RANKL-induced osteoclastogenesis. Therefore, the increased ERK activity may be responsible for enhanced survival and commitment of PKC-δ KO BMMs to the osteoclast lineage, increasing osteoclast numbers in an attempt to compensate for their reduced capacity for bone resorption.

In contrast to the in vitro data, bone histomorphometric analysis of osteoclasts in vivo showed that there was a slight reduction in osteoclast number in PKC-δ KO mice. The change is statistically significant and corresponds to a 10% reduction in osteoclast numbers, which, when coupled with resorption defects, accounts for the increased bone volume seen in the PKC-δ KO mice. This result suggests that the in vivo microenvironment may not be supportive of osteoclast formation. In vitro co-cultures of osteoblasts and BMMs show that PKC-δ KO osteoblasts can support osteoclastogenesis normally so osteoblasts may not be the limiting factor for in vivo osteoclastogenesis. However, PKC-δ KO mice display B-lymphocyte hyperproliferation and infiltration into many organs [22]. This finding is of great significance as B-lymphocytes contribute 64% of total osteoprotegerin (OPG) in the bone marrow [46]. In addition, B-lymphocyte deficient mice are osteoporotic [46]. This would suggest that the OPG: RANKL ratio may be skewed in favor of higher OPG levels in PKC-δ KO mice, inhibiting osteoclastogenesis in vivo. In combination with the resorption defect, reduced numbers of osteoclasts in vivo would have an additive effect on bone volume.

These studies have highlighted PKC-δ as a positive regulator of osteoclast bone resorption, which is in line with recent in vitro studies [36]. PKC-δ inhibition by Rottlerin was shown to reduce bone resorption by decreased acidification [36]. By comparison, we showed that acidification appeared to be normal in PKC-δ KO osteoclasts. However, we did observe changes in Src phosphorylation, an important regulator of bone resorption. In osteoclasts, Src not only has tyrosine kinase activity. but also acts as an adaptor molecule in osteoclast cytoskeletal reorganization, a function that does not require Src kinase activity [47]–[49]. Genetic knockout of Src in osteoclasts disrupts adhesion, cell motility and ruffled border formation [48]–[50]. As a result, Src deficient mice are osteopetrotic [8]. Src protein expression is normal in PKC-δ KO osteoclasts, and in alignment with its adaptor function, PKC-δ KO osteoclasts displayed normal cytoskeletal reorganization and adhesion. We found that Src416 phosphorylation is reduced at day 4 in PKC-δ KO osteoclasts compared to WT, indicating that PKC-δ is involved in Src416 phosphorylation during RANKL-induced osteoclastogenesis at the late stage.

Although the precise role of PKC-δ in Src signaling remains to be elucidated, it is possible that PKC-δ may regulate Src activity through PTP-α phosphatase activity [18], [20], [51]–[53]. Interestingly, the downstream target/substrate of Src, Cbl, may play a role in the PKC-δ KO osteoclast defect. Mutation of Cbl at the Src targeted phosphorylation site, Tyr-737 (in mouse Cbl), in osteoclasts produces a similar osteoclast phenotype to that of PKC-δ KO mice, namely enhanced osteoclastogenesis and reduced bone resorption but normal cytoskeletal reorganization [54]. Hence Cbl may be a candidate in PKC-δ-Src signaling. Cbl activates the phosphatidylinositol 3-kinase (PI3K)-Akt signaling pathway, which was recently shown to regulate ruffled border formation and vesicular transport in osteoclasts [55]. However, a recent study revealed that PKC-δ KO osteoclasts had a normal ruffled border and vesicular trafficking, although they do demonstrate reduced Cathepsin K secretion [37]. Surprisingly, we have found that the gene expression of cathepsin K is increased in PKC-δ KO osteoclasts. It is possible that PKC-δ KO osteoclasts increase cathepsin K mRNA expression in an attempt to compensate for impaired cathepsin K secretion as described by Cremasco et al. [37].

Having established a role for PKC-δ in osteoclast bone resorption, PKC-δ may represent a new therapeutic target for bone diseases. In support of previous studies [36], we have shown that Rottlerin inhibits bone resorption in vitro. Using the LPS-induced osteolysis model for inflammatory bone loss, which involves the stimulation of osteoclast activity by inflammatory cytokines such as TNF-α in an autocrine and paracrine manner [56]–[63], we further showed that PKC-δ KO mice are resistant to LPS-induced bone erosion. Furthermore, treatment with the PKC-δ inhibitor, Rottlerin, reduced LPS-induced bone erosion in WT mice. This shows that PKC-δ has a role in osteoclast bone resorption in vivo as supported by the presence of inactive osteoclasts in LPS-injected PKC-δ KO mice, reminiscent of bisphosphonate treated and Src-deficient osteoclasts [64], [65]. Therefore, PKC-δ has a role in inflammation-elicited bone destruction and is a potential therapeutic target in osteoclast-related diseases. This is particularly important since PKCs are in involved inflammation and cancer [9]–[11] and both are commonly associated with pathological osteolysis. Our results also indicate that PKC inhibitor Rottlerin with most specific action to PKC delta [66], has potential application for the inhibition of bone resorption and osteolysis. However, recent studies have questioned its specificity and efficacy to PKC-δ [67], [68], which might raise a challenge in its clinical application.

In conclusion, we have shown that PKC-δ KO mice exhibit an osteopetrotic phenotype, and resistance to LPS-induced osteolysis. Loss of PKC-δ leads to an intrinsic defect in osteoclastic bone resorption with altered signalling pathways and gene expression in osteoclasts, which is at least in part, due to an attempt to compensate for an intrinsic defect in osteoclast bone resorption. Collectively, this work contributes to our understanding of the role of PKC in osteoclast biology and might aid in the discovery of novel therapeutic avenues for bone diseases.
